# Histopathological Spectrum of the Upper Gastrointestinal Tract Endoscopic Biopsies at a Tertiary Hospital: A Descriptive Cross-sectional Study

**DOI:** 10.31729/jnma.8676

**Published:** 2024-07-31

**Authors:** Shraddha Koirala, Anu Khadka, Suzit Bhusal, Reshika Shrestha, Akanshya Prasai

**Affiliations:** 1 Department of Pathology, Nepal Medical College and Teaching Hospital, Kathmandu, Nepal; 2 Research and Development Unit, National Trauma Center, Kathmandu, Nepal; 3 Maharajgunj Medical Campus, Institute of Medicine, Kathmandu, Nepal; 4 Kathmandu Medical College and Teaching Hospital, Kathmandu, Nepal

**Keywords:** *biopsy*, *endoscopy*, *gastrointestinal*, *histopathology*, *neoplasm*

## Abstract

**Introduction:**

Upper gastrointestinal tract disorders are prevalent worldwide, encompassing neoplastic and non-neoplastic lesions like infections and inflammation. Endoscopic biopsies play a crucial role in diagnosis, treatment monitoring, and complication detection. Despite their routine use, comprehensive data on their histopathological spectrum is sparse. This study aimed to delineate this spectrum and assess the prevalence of non-neoplastic and neoplastic lesions in upper gastrointestinal tract endoscopic biopsies.

**Methods:**

This descriptive cross-sectional study at a tertiary care center analyzed upper gastrointestinal endoscopic biopsies from August 1, 2019, to July 31, 2021. After obtaining ethical clearance (reference number: 039-078/079), we collected all upper gastrointestinal endoscopic biopsies received during the two-year study period, excluding inadequate biopsies, resection specimens, therapeutic cases, and specific lesions. Histopathological examination was conducted using H&E, Giemsa, and Periodic acid-Schiff stains. Diagnoses were categorized into non-neoplastic and neoplastic lesions following WHO guidelines. Data were analyzed using SPSS 16.0 to determine the frequency of neoplastic and non-neoplastic cases.

**Results:**

Among 155 upper gastrointestinal biopsies, 124 (80%) were non-neoplastic (95% CI: 73.71-86.29%) and 31 (20%) were neoplastic (95% CI: 13.71-26.29%). Non-neoplastic lesions were predominantly chronic gastritis, with chronic active gastritis being the most frequent 34 (27.41%). Neoplastic lesions were mainly adenocarcinomas in the stomach 20 (64.51%) and squamous cell carcinomas in the esophagus 7 (22.58%).

**Conclusions:**

The prevalence of neoplastic lesions were found to be lower compared to the published literature and showed predominance of adenocarcinoma in upper gastrointestinal neoplastic lesions.

## INTRODUCTION

Upper Gastrointestinal (GI) tract disorders are some of the most frequently encountered cases in clinical practices all over the world.^1^ Lesions of the GI tract include neoplastic and non-neoplastic lesions like infections, inflammation, vascular disorders, physical and toxic injury etc.^2^ The endoscopic biopsies are also important for monitoring the course, determining the extent of a disease, as responses to therapy and for the early detection of complications.^3,4^ Endoscopic biopsy followed by histopathological evaluation is a relatively safe procedure and the current gold standard for assessing patients with gastrointestinal symptoms.^5^

The significant burden of upper GI disorders often present with nonspecific symptoms and require histopathological examination for accurate diagnosis.

Endoscopic biopsies are a routine diagnostic procedure, but there is insufficient data on the histopathological spectrum of these biopsies alone. Therefore, biopsy with histopathological study is essential for diagnosing upper GI lesions, prompting the need for this study.

The aim of this study was to find the histopathological spectrum of upper gastrointestinal tract endoscopic biopsies and the proportion of non-neoplastic and neoplastic lesions.

## METHODS

This descriptive cross-sectional study was conducted from 1^st^ August 2019 to 31^st^ July 2021 in Nepal Medical College Teaching Hospital (NMCTH). The data collection was started after ethical clearance from the Institutional Review Committee of the same institute with the reference number: 039-078/079. All upper GI endoscopic biopsies received in the Department of Pathology, NMCTH over the 2-year study period were the samples included in the study. Exclusion criteria are defined as follows: inadequate biopsies, resection specimens (gastrectomy and esophagectomy), and therapeutic endoscopy cases, which encompass the retrieval of foreign bodies, variceal ligation, and stricture dilatation. Additionally, all lesions of the mouth and pharynx, as well as duodenal biopsies below the second part, are excluded from the study. All the patients during the study period were included in the study and total sampling was done.

Data of all the patients who underwent endoscopy guided biopsy during the study period were retrieved from the department records and data collection was done retrospectively. All the endoscopic biopsy specimens received in the Department of Pathology were fixed in 10% buffered formalin, Paraffin embedded blocks were made and cutting and staining of the prepared slide with Hematoxylin and Eosin (H and E) was done. Additional sections were stained with Giemsa to observe Helicobacter pylori and Periodic Acid Schiff (PAS) stains wherever necessary. Slides stained with H and E and Giemsa were examined by the pathologist in detail and a diagnosis was made. Neoplastic lesions included adenocarcinoma, and squamous cell carcinoma. Non-neoplastic lesions include papillomas, inflammations and ulcers. The diagnosis was categorized into non neoplastic and neoplastic lesions. Tumors were diagnosed as per World Health Organization (WHO) histological classification of gastrointestinal tumors.^6^

Clinical details of the patients with respect to age, sex, site of the lesion, clinical features, endoscopic findings and histopathological diagnosis were recorded from the register maintained in the Department of Pathology.

The data retrieved from the department records were entered in Microsoft Excel and analyzed using Statistical Package for the Social Sciences (SPSS) version 16.0. Descriptive analysis was done where frequency and percentage were calculated for binary data. Point estimate at 95% Confidence Interval (CI) was also calculated.

## RESULTS

Among the 155 upper GI biopsies studied over a period of two years, the non-neoplastic lesions were 124 (80%; 95% Confidence Interval (CI): 73.71-86.29%) and neoplastic were 31 (20%; 95% CI: 13.71-26.29%). The site distribution of lesions is shown in Table 1.

**Table 1:** Distribution of the upper GI lesions (n= 155).

Site	n (%)
Esophagus	9 (5.80)
Gastro-esophageal junction	1 (0.64)
Stomach	126 (81.29)
Duodenum	19 (12.25)

Amongst the non-neoplastic lesions, 106 (85.48%) was in the stomach, 17 (13.70%) in the duodenum and 1 (0.80%) in the esophagus. The neoplastic lesions were distributed in stomach 20 (64.51%), esophagus 7 (22.58%), duodenum 3 (9.67%) and gastroesophageal junction 1 (3.22%). Biopsies with non-malignant findings from the stomach showed chronic gastritis; of which chronic active gastritis was present in 34 (27.41%) (Table 2).

**Table 2:** Histopathological spectrum of non-neoplastic lesions (n= 124).

Type	n (%)
**Esophagus**	
Papilloma	1 (0.80)
**Stomach**	
Chronic gastritis	
Mild	33 (26.61)
Moderate	19 (15.32)
Severe	15 (12.09)
Chronic active gastritis	34 (27.41)
H. pylori positive	25 (20.16)
Hyperplastic polyp	3 (2.41)
Gastric ulcer	1 (0.80)
Reactive gastropathy	1 (0.80)
**Duodenum**	
Ulcer	5 (4.03)
Non specific duodenitis	12 (9.67)

Malignant biopsies from the stomach and duodenum all showed adenocarcinoma accounting to 20 (64.51%) and 3 (9.67%) respectively whereas biopsies of the esophagus were all squamous cell carcinomas 7 (22.58%) (Table 3).

**Table 3:** Histopathological spectrum of neoplastic lesions (n = 31).

Type	n (%)
Esophagus	
Squamous cell carcinoma	7 (22.58)
Gastroesophageal junction	
Squamous cell carcinoma	1 (3.22)
Stomach	
Adenocarcinoma	20 (64.51)
Duodenum	
Adenocarcinoma	3 (9.67)

The gender distribution of total cases included male 67 (43%) and female 88 (57%). The distribution of the lesion in the 41-60 age group was 53 (34.19%) (Table 4).

**Table 4:** Distribution of patients according to age group (n= 155).

Age groups (years)	n (%)
6-20	17 (10.96)
21-40	42 (27.09)
41-60	53 (34.19)
61-80	39 (25.16)
>80	4 (2.58)

## DISCUSSION

The neoplastic lesions in this study were 31 (20%) which is comparatively less than 144 (33.25%) neoplastic lesions seen in the study by Memon et al. but higher than the 9.87% neoplastic lesions observed in another study conducted in India.^2,7^ Another study showed nonneoplastic lesions in 68.29% of the biopsy samples.^8^ Our study shows non-neoplastic lesions in 124 (80%) of the biopsy samples. The varying prevalence of neoplastic lesions observed in different studies may be attributed to several factors including population differences in genetics, sampling method, sample size, age category, and diagnostic criteria. Endoscopy offers a valuable method for visualizing the GI mucosa, often sufficient for diagnosing and managing specific clinical conditions. However, in many cases, tissue sampling during endoscopy is necessary. Examination of these specimens by a qualified pathologist is essential in the management of GI disorders. Common indications for upper GI endoscopy include dyspepsia, dysphagia, GERD, nausea, vomiting, occult bleeding, and cancer surveillance.^9^

The WHO classification of tumors of the digestive system, as outlined in the initial volume of the WHO series on tumor classification, 5^th^ edition, defines tumors on the basis of their molecular phenotype as their histological characteristics.^6^ However, histopathological classification remains the primary method for diagnosis in most cases.^6^ Most of the overall lesions were distributed to the stomach 126 (81.29%) followed by the duodenum 19 (12.25%) and then the esophagus with 9 (5.80%). The neoplastic lesions were distributed mostly to the stomach 20 (64.51%) followed by the esophagus 7 (22.58%) then the duodenum 3 (9.67%). The high frequency of endoscopically detected gastric lesions correlates with the study conducted in Pakistan by Memon et al. where the stomach was the most common location of endoscopic biopsy (51.3%), followed by the esophagus (39%), and the duodenum (9.7%) and the study conducted in Bangladesh by Islam et al. in 2014 where 73 (66.36%) were gastric, followed by 22 (20%) esophageal, and 15 (13.64%) duodenal biopsies.^2,10^ The frequency of gastric biopsies was higher in our study when compared to the other studies although consistent with the observed pattern where gastric biopsies were the most common. Interestingly, both referenced studies found esophageal biopsies to be more frequent in comparison to duodenal which is in contrast to our result of duodenal biopsies being more frequent than esophageal. The differing ratios may be due varying endoscopy practices as all three are single site studies, it may also be due to regional variations in disease prevalence.

Chronic gastritis was the most prevalent nonneoplastic lesion and adenocarcinoma was the predominant neoplastic lesion which correlates with the findings in the study by Hirachand et al. 2018 and Aparajita et al.^1,11^ This study found the most common non-neoplastic lesion to be chronic active gastritis with 34 (27.41%) followed closely by mild chronic gastritis with 33 (26.61%). Helicobacter pylori was present in 25 (20.16%) of cases. A study in India conducted under a similar methodology found Helicobacter pylori positivity in 59.4% of histopathological examinations of upper GI endoscopic biopsies.^12.^ These variations may be due to differences in patient demographics, or local environmental factors.

All neoplastic lesions of the stomach and duodenum were adenocarcinoma while those of esophagus were squamous cell carcinoma. A study by Sharma S et al. in India also found the majority of esophageal malignancies to be squamous cell carcinomas, and adenocarcinoma was found throughout the rest of the GI tract.^13^ Adenocarcinoma followed by squamous cell carcinoma represents the most frequent histological type of neoplastic lesion within the gastrointestinal system in our study. This finding is consistent with the results of another prospective study conducted in India.^5^ The distribution of neoplastic lesions observed aligns with established patterns of gastrointestinal cancer types and epidemiological trends.. The findings that all neoplastic lesions in the stomach and duodenum were adenocarcinomas and those in the esophagus were squamous cell carcinomas are explained by the histological predispositions of these regions. However, a study in the United States by Pohl and Welch found a rising trend in the incidence of adenocarcinoma in the esophagus.^14^ In contrast to our findings, which indicate a higher proportion of neoplastic lesions in the stomach followed by the esophagus, duodenum, and gastroesophageal junction, a study conducted in India reports a differing order with neoplastic lesions being most frequently detected in the esophagus, followed by the stomach and duodenum in descending order.^9^ The observed differences in the distribution of neoplastic lesions between our study and the study conducted in India could be due to various factors such as regional dietary habits, genetic variations, differences in regional healthcare practices and screening programs. The difference may indicate the need to consider local epidemiological trends when comparing cancer data across different populations.

The distribution of lesions varied across different age groups and between sexes. The largest incidence of upper GI endoscopic lesions was observed between 51-60 years of age by Aparajita et al. and in the 4th and 5th decade by Rashmi et al.^1,4^ Likewise, a study indicated that the majority of non-neoplastic cases occur in the 21-30 age group, while most neoplastic cases are observed in the 41-50 age group.^15^ These findings are in line with the result seen where the 41-60 years age group was observed to have the highest incidence of lesions. GI malignancies typically manifest at advanced stages due to their prolonged natural history, emphasizing the importance of early detection through increased endoscopic biopsies, particularly in older populations where incidence rates are higher. Therefore, any suspicion of GI pathology in middle to older age warrants endoscopic biopsy and histopathological analysis.^9^ Studies by Siddiqui, Aparajita et al. and Rashmi et al. found male predominance for upper GI lesions whereas our study shows female predominance (57%).^1,3,4^ Memon et al. 2015 shows female predominance in correlation with our study.^2^

The relatively small sample size and the singlecenter nature of this study could potentially introduce selection bias, thereby limiting the generalizability of the results to a larger, more diverse population. Expanding the study to multiple centers and including a larger, representative sample of patients would help provide a more comprehensive and generalizable understanding of the phenomenon under investigation. Prospective study for a longer duration with samples representing different parts of the population can give a more accurate picture. Longitudinal follow-up of patients, post-biopsy, could assess the impact of histopathological findings on disease progression and treatment outcomes.

## CONCLUSIONS

Though endoscopy proved invaluable for visualizing and managing various GI conditions, tissue sampling was often necessary for definitive diagnosis. We found that non-neoplastic lesions predominated, with chronic gastritis being the most prevalent histopathological finding, particularly in the stomach. Adenocarcinoma was the most common neoplastic lesion, primarily observed in the stomach and duodenum. Furthermore, studies among multiple centers and larger populations need to be performed to further validate and extend these observations.

## References

[1] Aparajita A, Mohanty RC, Sahu AA, Mohanty R, Satpathy PK, Bhuyan T (2016). Histomorphological Study of Upper GI Endoscopic Biopsies. Int J Health Sci Res..

[2] Memon F, Baloch K, Memon AA (2015). Upper gastrointestinal endoscopic biopsy.. Prof Med J..

[3] Siddiqui B A Study of Age-Wise Spectrum of Gastrointestinal Biopsies with Endoscopic Correlation a 5-Year Experience from a Tertiary Health Care Centre in North India.. Int J Pathol Clin Res.

[4] Rashmi K, Horakerappa MS, Karar A, Mangala G (2013). A STUDY ON HISTOPATHOLOGICAL SPECTRUM OF UPPER GASTROINTESTINAL TRACT ENDOSCOPIC BIOPSIES.. Int J Med Res Health Sci..

[5] Mishra R, Vahikar S, Shrivastava K, Mitra SK, Mishra V (2022). Histopathological spectrum of gastrointestinal lesions in patients undergoing gastrointestinal endoscopic biopsies-A Prospective study.. Tropical Journal of Pathology and Microbiology..

[6] Nagtegaal ID, Odze RD, Klimstra D, Paradis V, Rugge M, Schirmacher P (2020). The 2019 WHO classification of tumours of the digestive system.. Histopathology..

[7] Margaret Theresa J, Lavanya M, Gerard Rakesh J, Sultan Basha K (2020). Evaluating the spectrum of histomorphological patterns on endoscopic biopsy in patients with upper gastrointestinal tract disorders.. Tropical Journal of Pathology and Microbiology..

[8] Kaur M, Bhasin TS, Manjari M, Mannan R, Sharma S, Anand G (2018). Correlation between histopathological and endoscopic findings of non-malignant gastrointestinal lesions: an experience of a tertiary care teaching hospital from Northern India.. J Pathol Nep..

[9] Parikh BJ, Chilani AH, Nayak RC, Mistry KJ, Gediya PP, Parikh KB (2024). Histopathological spectrum of lesions of upper gastrointestinal tract - A study of endoscopic biopsies.. Asian J Med Sci..

[10] Islam SMJ, Mostaque Ahmed AS, Sahab Uddin Ahmad M, Hafiz S (2014). Endoscopic and Histologic Diagnosis of Upper Gastrointestinal Lesions, Experience in a Port City of Bangladesh.. Chatt Maa Shi Hosp Med Coll J..

[11] Hirachand S, Sthapit RR, Gurung P, Pradhanang S, Thapa R, Sedhai M (2018). Histopathological spectrum of upper gastrointestinal endoscopic biopsies.. J BP Koirala Inst Health Sci..

[12] Adlekha S, Chadha T, Krishnan P, Sumangala B (2013). Prevalence of helicobacter pylori infection among patients undergoing upper gastrointestinal endoscopy in a medical college hospital in kerala, India.. Ann Med Health Sci Res..

[13] Kumari K, Sharma S, Sharad S, Shah G, Neelam (2020). Histopathological spectrum of lesions in gastrointestinal endoscopic biopsy: A prospective study of 500 cases.. Ind J Pathol Oncol..

[14] Pohl H, Welch HG (2005). The role of overdiagnosis and reclassification in the marked increase of esophageal adenocarcinoma incidence.. J Natl Cancer Inst..

[15] Kumawat DN, Shah DS, Goswami DH (2021). A study of histopathological spectrum of gastrointestinal tract lesions in a tertiary care centre.. Int J Clin Diagn Pathol..

